# The Olfactory Receptor Olfr25 Mediates Sperm Dysfunction Induced by Low-Dose Bisphenol A through the CatSper-Ca^2+^ Signaling Pathway

**DOI:** 10.3390/toxics12060442

**Published:** 2024-06-20

**Authors:** Jing Gu, Ning Zhang, Xiao Jiang, Lei Zhu, Yixia Lou, Shengqi Sun, Li Yin, Jinyi Liu

**Affiliations:** 1 State Key Lab of Trauma and Chemical Poisoning, Key Lab of Medical Protection for Electromagnetic Radiation, Ministry of Education of China, Institute of Toxicology, College of Preventive Medicine, Army Medical University, Chongqing 400038, China; jinggu@tmmu.edu.cn (J.G.); zhang.ning0903@163.com (N.Z.); jx3136@tmmu.edu.cn (X.J.); fuermosi76@163.com (L.Z.); lyxttkx5@163.com (Y.L.); s18737351892@163.com (S.S.); 2 Chongqing Key Lab of Medicinal Chemistry and Molecular Pharmacology, Chongqing University of Technology, Chongqing 400054, China

**Keywords:** bisphenol A, low-dose, sperm function, Olfr25, CatSper, calcium ion concentration

## Abstract

Bisphenol A (BPA), a typical endocrine disruptor, is known to have various adverse effects on the male reproductive system. However, the toxic effects and mechanisms of low-dose BPA have not yet been fully explored. In this study, male Kunming mice were orally administered low-dose BPA (0.03, 0.3 and 3 mg/kg/d) for ten consecutive weeks. Pathological sections of testicular tissue showed no significant morphological differences after BPA exposure. An analysis of the functional parameters of sperm revealed that exposure to low-dose BPA significantly decreased sperm motility, chemotaxis, and the acrosome reaction. An in vitro BPA exposure model combined with an omics data analysis showed that the olfactory receptor-related pathway was significantly enriched after BPA treatment. Subsequent experiments verified the reduced mRNA level of a novel olfactory receptor gene, *Olfr25*, in vivo and in vitro exposure models. Meanwhile, exposure to low-dose BPA reduced the intracellular calcium ion concentration and the mRNA levels of pore-forming subunits of the CatSper channel in sperm. Importantly, the knockdown of Olfr25 inhibited calcium ion levels and CatSper subunit expression in GC-2 cells. Olfr25 overexpression attenuated the BPA-induced downregulation of CatSper subunit expression in GC-2 cells. These findings indicate that Olfr25 might participate in low-dose BPA-induced sperm dysfunction by affecting the CatSper-Ca^2+^ signaling pathway. This study reveals a new mechanism underlying the effects of low-dose BPA on sperm function and provides a reference for assessing the safety of low-dose BPA exposure.

## 1. Introduction

Bisphenol A (BPA) is an industrial plasticizer extensively applied in the manufacture of polycarbonate plastics and epoxy resins for many consumer products [1,2]. The widespread use of BPA, together with its ability to leach into water and food products, results in ubiquitous human exposure to BPA [3]. The predominant exposure route for humans is through dietary ingestion, while transdermal and respiratory routes are also considered common and non-negligible [4]. BPA has been detected in various human body fluids and tissues, such as serum, plasma, urine, amniotic fluid, breast milk, and adipose tissue [3,5]. Due to its polycyclic phenolic structure, BPA is considered an endocrine disruptor (ED) and to be involved in various diseases, for example, reproductive abnormalities, metabolic diseases, developmental disorders, and cancers [1,6,7,8,9]. Growing evidence supports the notion that BPA, as a reproductive toxicant, impairs spermatogenesis by eliciting deleterious effects on spermatozoa, such as inducing mitochondrial dysfunction and oxidative/apoptotic damage [10,11]. Moreover, population studies have shown a negative correlation between semen parameters and urinary BPA levels [11].

Once ejaculated into the female reproductive tract, mammalian spermatozoa need to undergo various physiological processes to become capable of reaching and fertilizing the oocyte, such as chemotaxis, capacitation, hyperactivation, and the acrosome reaction [12]. Calcium (Ca^2+^), as an intracellular second messenger, functions as a central regulator in these fundamental processes needed for fertilization [13,14]. Ca^2+^ deficiency in spermatozoa is associated with male infertility [15]. The Cation Channel of Sperm (CatSper) is a sperm-specific Ca^2+^ channel located in the membrane of the flagellar principal piece, and it controls the intracellular Ca^2+^ concentration ([Ca^2+^]i) [16,17]. CatSper is a heterotetrameric complex composed of four separate pore-forming α-subunits (CatSper1–4) and six ancillary subunits (CatSperβ, γ, δ, ɛ, and ζ, and EFCAB9) [18]. Knocking out any of the four CatSperα subunits in mice causes male infertility [19,20]. Patients with asthenoteratozoospermia have also been found to have insertion/deletion mutations in *Catsper1* or *Catsper2* genes [21,22].

Olfactory receptors (ORs) are classified as G protein-coupled receptors, and they consist of seven α-helical transmembrane domains linked by three extracellular and three intracellular loops with an extracellular N-terminus and an intracellular C-terminus [23,24]. ORs were first identified in the rat olfactory epithelium, and they are primarily responsible for smell perception through the recognition of specific odorous ligands [23,25]. However, accumulating studies have reported the widespread ectopic expression of ORs in various non-olfactory tissues, such as the testis, heart, kidney, liver, prostate, and lung, and their ectopic expression is involved in several key physiological processes [26,27]. Notably, testicular tissue possesses the largest number of ectopically expressed OR transcripts [28]. ORs are localized in pre- and post-meiotic germ cells, as well as in the head, tail, and midpiece of mature spermatozoa, and they are crucial for the maturation, migration, and chemotaxis of sperm [26,29]. For instance, human OR17-4 (hOR17-4), located in the spermatozoon midpiece, is activated by the odorant bourgeonal, thus eliciting pronounced Ca^2+^ fluxes and chemotaxis, the activation of which is coupled to a cAMP-mediated signaling cascade [30,31]. Mouse OR23 (MOR23), expressed in round spermatids during stages VI–VIII of spermatogenesis, modulates the flagellar configuration and chemotaxis by inducing an influx of cellular Ca^2+^ [32]. A human OR (OR51E2), identified in the flagella, midpiece, and acrosomal cap of spermatozoa, facilitates the migration of sperm upon stimulation by specific short-chain fatty acids [29,33].

In this work, we investigated the effects of low-dose BPA on the male reproductive system and the underlying regulatory mechanism using in vivo and in vitro BPA exposure models combined with omics data analysis. We identified a novel olfactory receptor gene, *Olfr25*, which might mediate low-dose BPA-induced sperm function impairment through the CatSper-Ca^2+^ signaling pathway.

## 2. Materials and Methods

### 2.1. Cell Culture and Treatment

GC-2 cells derived from mouse spermatocytes were purchased from the American Type Culture Collection (ATCC). The cells were grown in high-glucose DMEM (Sigma-Aldrich, St. Louis, MO, USA) supplemented with 10% fetal bovine serum (FBS) (Gibco, Grand Island, NY, USA) and maintained in an incubator at 37 °C with a 5% CO_2_ concentration. BPA (Sigma-Aldrich, St. Louis, MO, USA) was dissolved in DMSO for use as a stock solution.

### 2.2. Animals and BPA Exposure Protocol

Adult male Kunming mice (8 weeks old), weighing 32–35 g, were acquired from the Experimental Animal Center of the Army Medical University. The animals were housed under constant conditions (23 ± 2 °C, 55 ± 5%  relative humidity, and a 12 h light/12 h dark cycle) and allowed free access to standard mouse chow (Cooperative Medical Biological Engineering Co., Ltd., Nanjing, China) and distilled water. Prior to experimental procedures, the mice were allowed to acclimate for one week. Based on systemic toxicity studies, the Food and Drug Administration (FDA) established that the no-observed-adverse-effect level (NOAEL) of BPA for humans is 5 mg/kg/d [34]. Accordingly, the mouse equivalent dose is 30 mg/kg/d, determined through dose conversion based on the normalization of the dose-to-body surface area [35,36,37]. Thus, 0.03, 0.3, and 3 mg/kg/d (representing 1/1000, 1/100, and 1/10 of the dose equal to NOAEL, respectively) were chosen as the low-exposure doses of BPA. The mice (8 per group) were gavaged with either BPA at different doses dissolved in corn oil (0.1 mL/10 g body weight) or corn oil alone as a control for ten consecutive weeks. Subsequently, the mice were anesthetized and euthanized through cervical dislocation, and their testes and epididymides were removed. All experiments were conducted in strict accordance with the guidelines for the use and management of laboratory animals approved by the Ethical Committee of Army Medical University (approval no. AMUWEC2020074).

### 2.3. Testicular Pathological Analysis

The testes were quickly dissected and fixed in Bouin’s fluid. After dehydration, the fixed tissues were embedded in paraffin and then sliced at a thickness of 5 µm using a microtome. These paraffin sections were dewaxed, rehydrated, and stained with hematoxylin and eosin (HE). Histopathological alterations in the testicular tissues were observed and photographed by using a light microscope (Olympus DP73, Tokyo, Japan).

### 2.4. Sperm Motility Analysis

The measurement of sperm motility was conducted by following a previously described procedure [38]. Briefly, the epididymides were removed and transferred to 500 μL of human tubal fluid (HTF) medium (Millipore, Burlington, MA, USA). Two cuts were made in each cauda, allowing sperm to disperse into the medium. The tissue was removed, and the suspension was mixed gently for 10 min at 37 °C. Aliquots (20 μL) of sperm suspensions were detected using a computer-assisted sperm analysis (CASA) system (Suiplus SSA-II, Beijing, China). The motility characteristics of at least 200 sperm were assessed for each sample in five fields.

### 2.5. Sperm Chemotaxis Assay

Sperm chemotaxis was evaluated using a microfluidic chip (CapitalBio Corporation, Beijing, China). Prior to performing the assay, the microfluidic device was treated in a plasma generator for hydrophilic surface modification, and then it was rinsed with HTF medium containing 0.25% (*w*/*v*) bovine serum albumin (BSA). To obtain cumulus cells, female Kunming mice were superovulated via intraperitoneal injection of 10 IU pregnant mare serum gonadotropin (PMSG) (Ningbo Second Hormone Factory, Ningbo, China), followed by a 48 h interval and injection of 10 IU human chorionic gonadotropin (HCG) (Ningbo Second Hormone Factory, Ningbo, China). Fourteen hours after the HCG injection, the mice were anesthetized and sacrificed through cervical dislocation. Then, the oviducts were dissected and fat tissues were carefully removed. Oocytes and the surrounding cumulus cells were extracted from the oviductal ampullae and placed in HTF medium supplemented with 3 mg/mL hyaluronidase. After digestion for 5 min at 37 °C, the oocytes were removed, and the cumulus cells were resuspended in HTF medium containing 10% FBS. To form a chemoattractant gradient, the suspensions were seeded in one of the outlet pools of the microfluidic chip with a mineral oil overlay to prevent evaporation for 24 h at 37 °C. During the incubation period, the fluid in the microfluidic chip avoided turbulence. The male mice gavaged with BPA at different doses were sacrificed through cervical dislocation under anesthesia. The sperm collected from the epididymides were subjected to capacitation via incubation at 37 °C for 45 min in a humidified 5% CO_2_ incubator. Then, 2.5 × 10^4^ sperm were added to the inlet pool of the microfluidic chip and reached the bifurcation consistently after 15 min of swimming. The CASA system was used to detect sperm passing through two outlet pools of the microfluidic chip, and the percentage of chemotactic sperm was calculated.

### 2.6. Assessment of the Acrosome Reaction

To assess the acrosome reaction, chlortetracycline (CTC) staining was conducted following a previously described method [39,40]. After incubation for 45 min at 37 °C, 20 µL aliquots of capacitated spermatozoa were stimulated with 20 μL of 150 μM progesterone to induce the acrosome reaction. Then, 20 μL of the spermatozoal suspension was stained with an equal volume of 250 μM CTC solution for 15 min at 37 °C and incubated with 0.5 μL Hoechst 3342 solution for 2 min. The samples were fixed by mixing with 12.5% glutaraldehyde. The mixture was smeared on a microscope slide and observed with a fluorescence microscope (Olympus BX53F, Tokyo, Japan). A minimum of 200 spermatozoa were counted to assess the CTC staining patterns. Decreased or no fluorescence over the head of the spermatozoa was indicative of the acrosome-reacted pattern.

### 2.7. Microarray and Data Analysis

Microarray analysis was performed with GMINIX (Shanghai, China) using GeneChip^®^ Mouse Gene 2.0 ST Arrays (Affymetrix, Santa Clara, CA, USA). Briefly, the total RNA was extracted from GC-2 cells exposed to 80 μM BPA for 48 h using Trizol reagent (Takara, Dalian, China) and transcribed into cDNA. Following fragmentation and biotin labeling, the cDNA samples were then subjected to hybridization on a microarray chip. After washing and staining, the arrays were scanned to acquire raw data. The data were subsequently normalized and analyzed using GeneSpring GX v11.5.1 software (Agilent Technologies, Santa Clara, CA, USA). Differentially expressed genes (*p*-value cutoff of 0.05 and fold-change cutoff of 1.5) were utilized to conduct Gene Ontology (GO) and Kyoto Encyclopedia of Genes and Genomes (KEGG) enrichment analyses using DAVID (https://david.ncifcrf.gov/, accessed on 20 April 2021) [41].

### 2.8. RNA Extraction and qRT-PCR Analysis

The sperm pellets collected from the epididymides were incubated for 10 min with somatic cell lysis buffer containing 0.1% SDS and 0.5% Triton X-100 to remove potential contamination from somatic cells. Following lysis, the spermatozoa were centrifuged at 800 rpm for 5 min and then rinsed twice with PBS. The total RNA was isolated from the spermatozoa and GC-2 cells using TRIzol reagent (Takara, Dalian, China) following the manufacturer’s protocol. The PrimeScript^®^ RT Reagent Kit (Takara, Dalian, China) was used to generate the cDNA. qRT-PCR was performed using the SYBR Premix Ex Taq Kit (TaKaRa, Dalian, China) to quantify the relative mRNA expression levels based on the 2^−ΔΔCt^ method. The mRNA levels of each sample were normalized to the levels of β-actin. The primer sequences used in this assay are provided in Table S1.

### 2.9. Cell Transfection

To knock down Olfr25, small interfering RNA (siRNA) specific for Olfr25 knockdown and negative control siRNA were designed and synthesized by Sangon Biotech (Shanghai, China). To overexpress Olfr25, the gene encoding Olfr25 was chemically synthesized by Tsingke Biotech (Beijing, China) and cloned into a TK-PCDH-copGFP-T2A-Puro plasmid. Lipofectamine 3000 (Invitrogen, Carlsbad, CA, USA) was employed to transfect siRNA or plasmids into GC-2 cells according to the manufacturer’s protocol. The cells stably transfected with Olfr25 plasmids were subjected to selection by treatment with puromycin (Beyotime). The sequences of the siRNA oligonucleotides in the experiment are provided in Table S1.

### 2.10. Assessment of Intracellular Calcium Ion Levels

[Ca^2+^]i levels in the spermatozoa and GC-2 cells were evaluated using the fluorescent calcium probe Fluo-4 AM (Beyotime, Shanghai, China) based on a previous procedure [38]. Briefly, capacitated spermatozoa were loaded with 1 μM Fluo-4 AM in the dark for 30 min at 37 °C and were subsequently washed twice with HBSS (Beyotime, Shanghai, China). After centrifugation at 800 rpm for 5 min, the Fluo-4-loaded spermatozoa were resuspended with HTF medium, and 20 μL of the spermatozoal suspension was immediately placed on a microscope slide and covered with a coverslip. The intensity of fluorescence was examined using a fluorescence microscope (Olympus BX53F, Tokyo, Japan). Olfr25 siRNA-transfected GC-2 cells were trypsinized and resuspended in HBSS. The cells were then stained with Fluo-4 AM following the procedure described above. The fluorescence of Fluo-4 was measured using a FACSCalibur flow cytometer (BD Biosciences, San Jose, CA, USA). FlowJo software V10 (TreeStar, Ashland, OR, USA) was used to analyze the intensity of fluorescence. The experiments were conducted three times.

### 2.11. Western Blot Analysis

The total protein of the GC-2 cells with differential Olfr25 expression was extracted with a RIPA lysis solution mixed with a protease inhibitor (Beyotime, Shanghai, China). Following 30 min of incubation on ice, the lysates were centrifuged at 12,000 rpm for 20 min. Protein concentrations were measured using the BCA protein assay kit (Beyotime, Shanghai, China). SDS-PAGE was used to isolate the denatured protein samples, which were then transferred onto PVDF membranes (Millipore, Bedford, MA, USA). After blocking with 3% BSA in TBST, the blots were probed with specific primary antibodies and then horseradish peroxidase (HRP)-conjugated secondary antibody. The antibodies used in this study included CatSper1 (1:1000, orb234792, Biorbyt, Cambridge, UK), CatSper2 (1:1000, orb156278, Biorbyt, Cambridge, UK), CatSper3 (1:1000, orb520441, Biorbyt, Cambridge, UK), CatSper4 (1:1000, DF9351, Affinity Biosciences, Changzhou, China), β-actin (1:1000, AF0003, Beyotime, Shanghai, China), and HRP-goat anti-rabbit or anti-mouse IgG (H+L) (1:1000, A0208 or A0216, Beyotime, Shanghai, China). An enhanced chemiluminescence reagent (Invigentech, Irvine, CA, USA) was used to measure the protein bands. ImageJ 1.52v software was used to perform quantitative analysis of the indicated proteins.

### 2.12. Edu Staining Assay

An Edu assay was conducted using the Cell-Light™ EdU Apollo^®^567 In Vitro Imaging Kit (RiboBio, Guangzhou, China) following the manufacturer’s protocol. Briefly, GC-2 cells were cultured in 96-well plates overnight and transfected with Olfr25 siRNA. The cells were incubated with 50 μM of Edu solution for 2 h, followed by fixation using 4% formaldehyde for 30 min at 37 °C. Subsequently, the cells were neutralized using glycine for 5 min and treated with 0.5% Triton X-100 for 10 min. After being washed with PBS thrice, the cells were incubated in Apollo staining solution and counterstained with Hoechst 33342 reaction solution for 30 min. All images were observed using a fluorescence microscope (Olympus BX53F, Tokyo, Japan). Five visual fields were randomly selected in each sample image.

### 2.13. Cell Cycle Analysis

GC-2 cells were placed in 6-well plates and transfected with Olfr25 siRNA after serum deprivation for 12 h. The cells were harvested 36 h after transfection and fixed overnight in 70% ethanol at 4 °C. The fixed cells were washed three times with PBS and stained with 25 µg/mL of propidium iodide (PI) (Beyotime, Shanghai, China) solution containing 100 µg/mL of RNase for 30 min in the dark. DNA content was measured using a FACSCalibur (BD Biosciences, San Jose, CA, USA). Cell cycle status was analyzed using FlowJo software V10 (TreeStar, Ashland, OR, USA).

### 2.14. Statistical Analysis

All data in this study were analyzed using GraphPad Prism 9.0.0 software (San Diego, CA, USA). Data are presented as the mean ± standard deviation (SD). Statistical differences between two groups were analyzed using the Student’s *t*-test. Differences among more than two groups were analyzed using a one-way ANOVA with Dunnett’s multiple comparison test. *p* < 0.05 was considered statistically significant.

## 3. Results

### 3.1. Low-Dose BPA Exposure Induced Sperm Dysfunction in Mice

**Figure 1:**
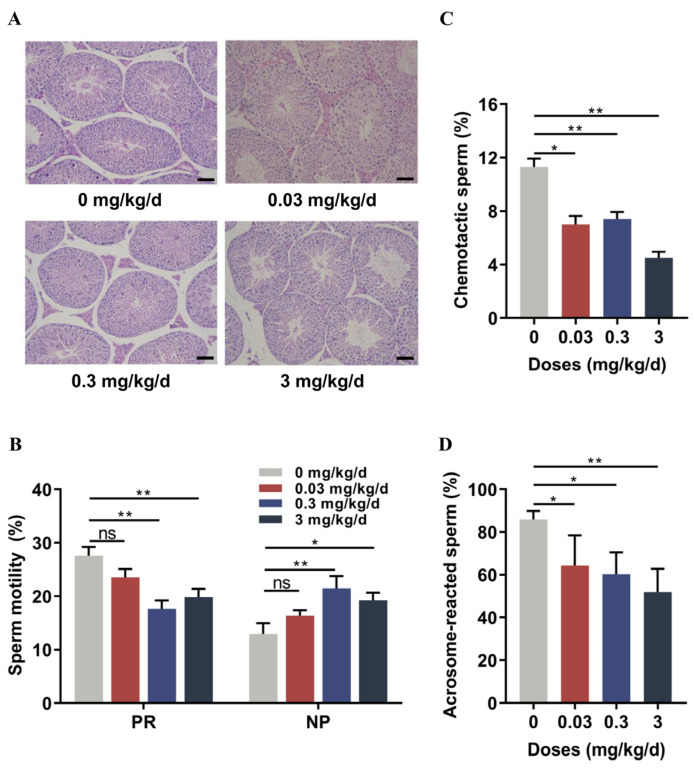
Low-dose BPA impaired the motility, chemotaxis, and acrosome reaction of mouse sperm. (**A**) Representative histopathological images of mouse testicular tissues with HE staining (n = 3). Scale bars are 50 µm. (**B**,**C**) Sperm functional parameters, including sperm motility (**B**) and chemotaxis (**C**), measured using CASA analysis (n = 8). NP, non-progressive motility. PR, progressive motility. (**D**) Acrosome reaction induced by progesterone (n = 5). All data are presented as the mean ± SD. ns, not significant. * *p* < 0.05, ** *p* < 0.01.

To explore the effect of low-dose BPA on the reproductive system of male mice, we administered BPA to adult Kunming mice at concentrations of 0.03, 0.3, and 3 mg/kg/d for ten weeks. After low-dose BPA exposure, the final body weights and testis weights of the mice were not significantly different from those of the control group (Figure S1). Pathological examination of testicular tissue was performed after BPA exposure. The low-dose BPA-treated mice showed highly convoluted seminiferous tubules and clearly organized spermatogenic cells similar to those observed in the control mice (Figure 1A); this indicated that low-dose exposure caused no significant morphological differences in the testes. Furthermore, the sperm concentration and motility in the epididymis were analyzed using the CASA system. The low-dose BPA-treated mice exhibited no significant change in sperm concentration (Figure S2) but a decrease in sperm motility (Figure 1B). Only at a dose of 0.03 mg/kg/d was no statistically significant change observed in sperm motility. Notably, progressive sperm motility at doses of 0.3 and 3 mg/kg/d showed significant decreases compared with that of the control group. Meanwhile, non-progressive sperm motility increased predominately at doses of 0.3 and 3 mg/kg/d.

Sperm can sense chemoattractants secreted from the oocyte and its surrounding cumulus cells, which drives chemotactic behavior to orient toward the oocyte [42]. To determine whether low-dose BPA affects sperm chemotaxis, chemotaxis assays were conducted using chemoattractants from the cumulus cells of female mice. These results showed that the sperm chemotactic rate declined significantly in all groups treated with BPA, as compared with the control group (Figure 1C). The acrosome reaction is a prerequisite for sperm to penetrate the zona pellucida and fuse with the oocyte’s plasma membrane [43]. Here, the acrosome reaction of sperm in response to progesterone stimulation was examined in the low-dose BPA-treated mice. Compared with the control group, progesterone-induced AR in the exposure groups declined significantly in a dose-dependent manner (Figure 1D and Figure S3). Taken together, these results show that exposure to low-dose BPA significantly impaired the function of sperm.

### 3.2. Low-Dose BPA Exposure Inhibited the Expression of Olfactory Receptor Olfr25 in GC-2 Cells and Sperm

**Figure 2:**
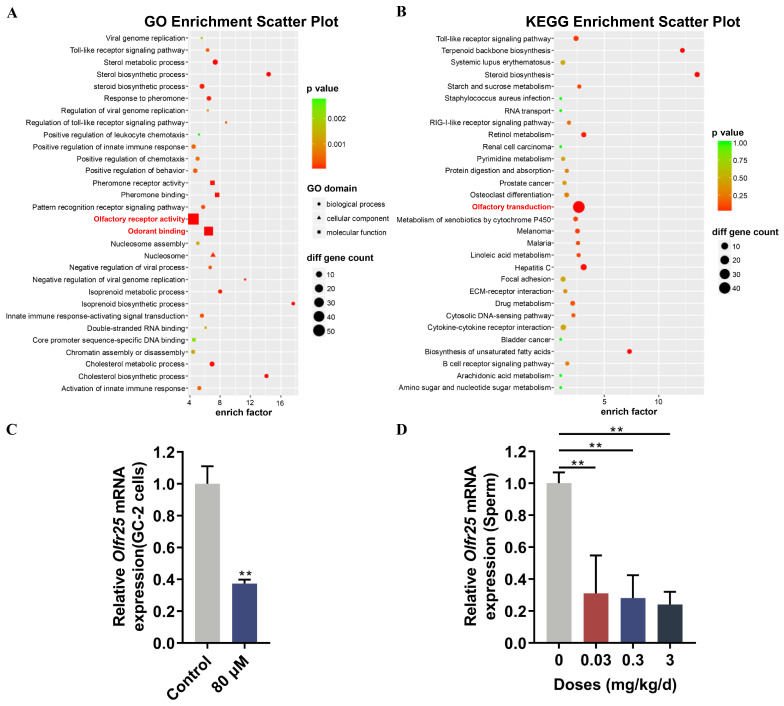
The effect of low-dose BPA exposure on the expression of olfactory receptor Olfr25. (**A**,**B**) GO and KEGG enrichment analyses of differentially expressed genes (DEGs) in GC-2 cells following exposure to BPA. The top 30 GO terms (**A**) and enrichment pathways (**B**) are illustrated in bubble plots. The enriched terms related to the olfactory receptor are highlighted in red. (**C**,**D**) The mRNA expression level of olfactory receptor gene *Olfr25* in GC-2 cells and mouse sperm after BPA exposure was validated using qRT-PCR (n = 3). All data are presented as the mean ± SD. ** *p* < 0.01.

To elucidate the mechanism of low-dose BPA-induced sperm function impairment, we established an in vitro BPA exposure model using mouse spermatocyte-derived GC-2 cells. Based on the IC50 value of BPA (160 μM) determined in our previous studies [44], we chose to expose GC-2 cells to 80 μM BPA for 48 h and subsequently performed microarray analysis. GO and KEGG enrichment analyses were subsequently performed for differentially expressed genes (DEGs). The GO annotations of biological processes showed that the DEGs mainly participated in olfactory receptor activity and odorant binding (Figure 2A and Table S2). The KEGG pathway annotation showed that the DEGs were markedly enriched in the olfactory transduction pathway (Figure 2B and Table S3). We further verified the expression of olfactory receptor genes using qPCR. The results indicated that BPA treatment could significantly reduce the expression of olfactory receptor gene *Olfr25* in GC-2 cells (Figure 2C). Consistently, the expression of *Olfr25* in sperm was downregulated, and the decrease exhibited dose dependence after low-dose BPA exposure (Figure 2D). Given the importance of olfactory receptors in controlling sperm functions, such as capacitation, motility, and chemotaxis [29,45], we speculated that olfactory receptor Olfr25 might be involved in the regulation of sperm dysfunction induced by low-dose BPA.

### 3.3. Low-Dose BPA Exposure Reduced Calcium Ion Levels and CatSper Subunit Expression in Sperm

**Figure 3:**
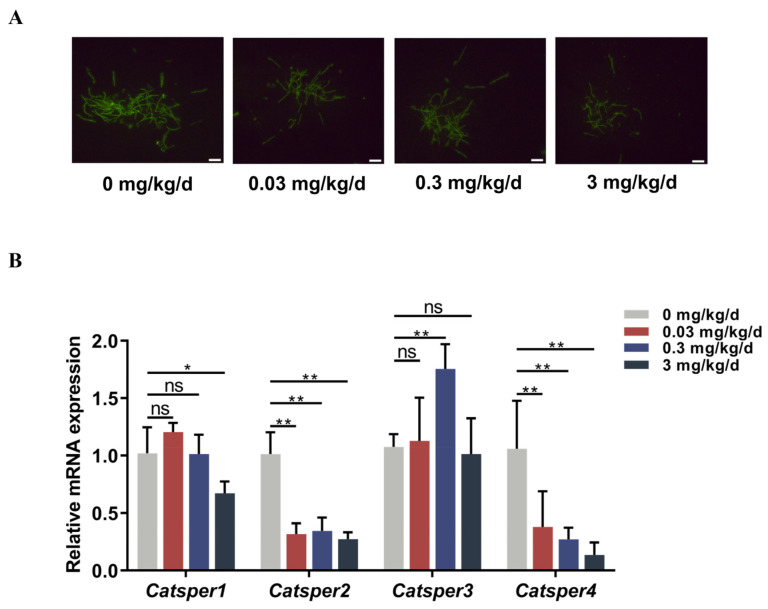
The effect of low-dose BPA exposure on calcium ion levels and the expression of CatSper channel genes in mouse sperm. (**A**) Representative fluorescence images of mouse sperm stained with Ca^2+^ probe Fluo-4 AM. Scale bars are 20 µm. (**B**) The mRNA expression levels of *Catsper1–4* in sperm were detected using qRT-PCR (n = 3). All data are presented as the mean ± SD. ns, not significant. * *p* < 0.05, ** *p* <0.01.

The Ca^2+^ concentration in sperm is pivotal in controlling motility patterns, chemotaxis, and the acrosome reaction [13]. To assess whether low-dose BPA affects [Ca^2+^]i in sperm, we monitored Ca^2+^ levels after BPA exposure using a Fluo-4 probe. As shown in Figure 3A, the fluorescence intensity of Fluo-4 significantly decreased with the increase in BPA dose, indicating that low-dose BPA exposure reduced [Ca^2+^]i in sperm. The CatSper ion channel, which is a sperm-specific voltage-gated Ca^2+^ channel, mediates a variety of sperm functions [16]. To clarify whether the decrease in [Ca^2+^]i in BPA-treated mice resulted from changes in *Catsper* gene expression, we analyzed the expression levels of the four pore-forming CatSper subunits using qPCR. The results showed that the mRNA levels of the pore-forming CatSper subunits decreased in the exposure groups compared with the control group (Figure 3B). Specifically, *Catsper2* and *Catsper4* were downregulated dramatically after BPA exposure. Thus, these results indicated that low-dose BPA downregulated [Ca^2+^]i in sperm by decreasing the expression of CatSper subunits.

### 3.4. Olfr25 Knockdown Inhibited Calcium Ion Levels and CatSper Subunit Expression in GC-2 Cells

**Figure 4:**
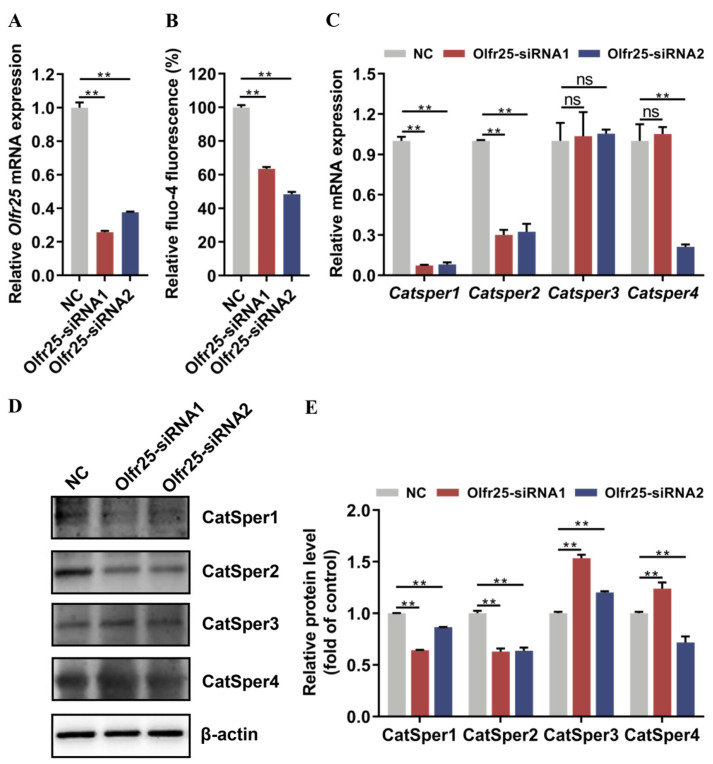
The effect of Olfr25 knockdown on calcium ion levels and the expression of CatSper channel genes in GC-2 cells. (**A**) RT-qPCR was used to measure the mRNA expression levels of *Olfr25* (n = 3). (**B**) Intracellular calcium ion concentration was detected after Olfr25 knockdown (n = 3). (**C**) The mRNA expression levels of *Catsper1–4* in GC-2 cells after Olfr25 knockdown were detected using qRT-PCR (n = 3). (**D**) The protein expression levels of CatSper1–4 in GC-2 cells after Olfr25 knockdown were detected using Western blot analyses. (**E**) Quantitative analysis of the indicated proteins using Image J software. All data are presented as the mean ± SD. ns, not significant. ** *p* < 0.01.

To determine the role of Olfr25 in low-dose BPA-induced sperm dysfunction, we opted to utilize GC-2 cells to construct a cell model with Olfr25 knockdown. Considering the previous experimental results, we subsequently investigated the potential involvement of Olfr25 in the regulation of the CatSper-Ca^2+^ signaling pathway in vitro. The knockdown efficiency of *Olfr25* was verified using qRT-PCR (Figure 4A). Flow cytometry was employed to measure [Ca^2+^]i after Olfr25 knockdown. The results proved that the knockdown of Olfr25 dramatically decreased [Ca^2+^]i in GC-2 cells (Figure 4B). The expression levels of the pore-forming CatSper subunits after Olfr25 knockdown were further detected through qRT-PCR and Western blot analysis. We found that the expression levels of the CatSper1, CatSper2, and CatSper4 subunits but not the CatSper3 subunit were significantly downregulated when Olfr25 was knocked down (Figure 4C–E). To further confirm the other potential roles of Olfr25 in GC-2 cells, an Edu assay and cell cycle analysis were conducted after Olfr25 knockdown. There were no significant changes in cell proliferation or cell cycle progression after Olfr25 knockdown (Figure S4), indicating that Olfr25 does not play a requisite role in cell proliferation and cell cycle progression in GC-2 cells. Overall, these results suggested that Olfr25 might control the changes in intracellular Ca^2+^ levels by regulating the expression of CatSper subunits.

### 3.5. Olfr25 Overexpression Attenuated the BPA-Induced Downregulation of CatSper Subunit Expression in GC-2 Cells

**Figure 5:**
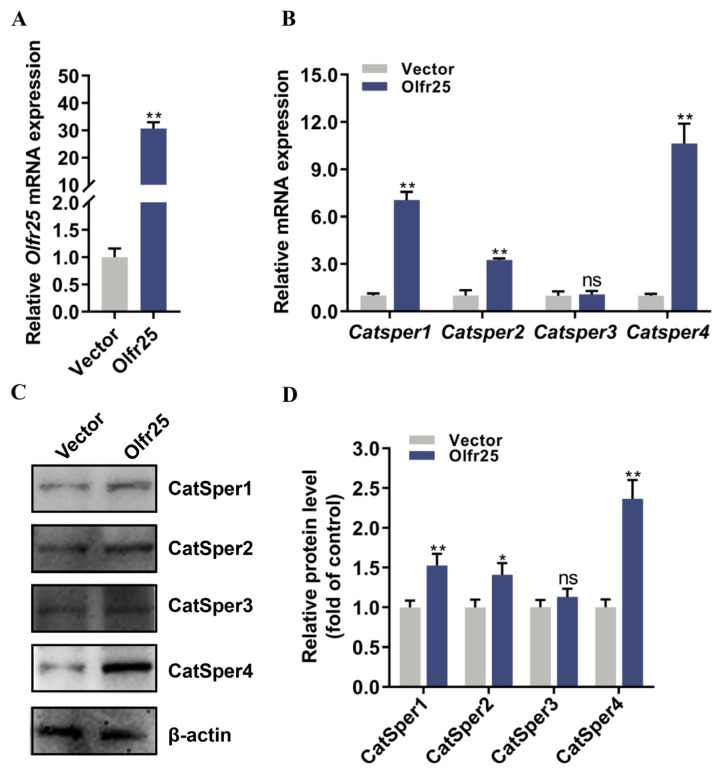
The effect of Olfr25 overexpression on the expression of CatSper channel genes in GC-2 cells. (**A**) RT-qPCR was used to measure the mRNA expression levels of *Olfr25* in GC-2 cells after Olfr25 overexpression (n = 3). (**B**) The mRNA expression levels of *Catsper1–4* in GC-2 cells after Olfr25 overexpression were detected using qRT-PCR (n = 3). (**C**) The protein expression levels of CatSper1–4 in GC-2 cells after Olfr25 overexpression were detected using Western blot analysis. (**D**) Quantitative analysis of the indicated proteins using Image J software. All data are presented as the mean ± SD. ns, not significant. * *p* < 0.05, ** *p* < 0.01.

**Figure 6:**
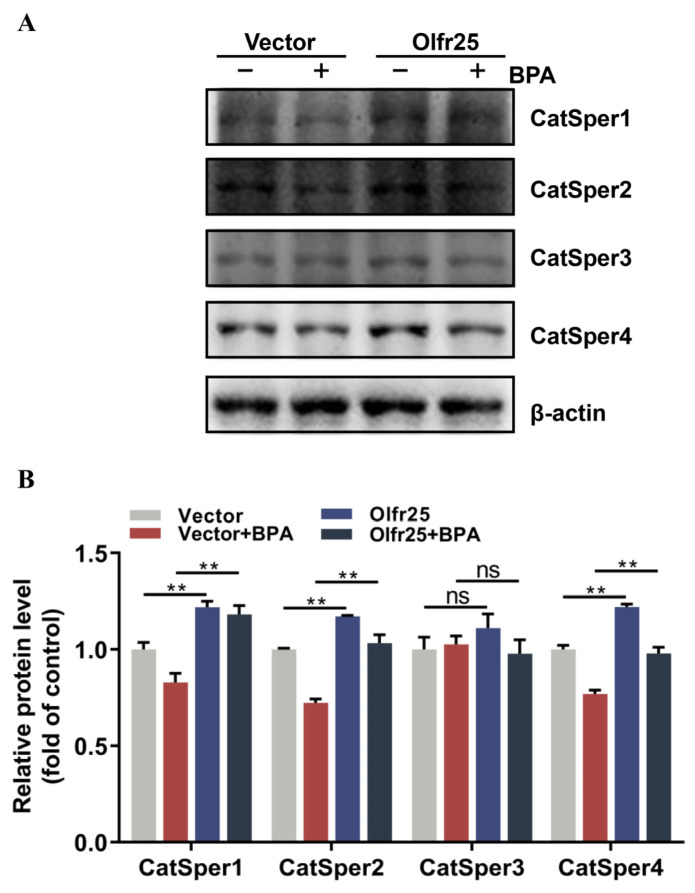
Olfr25 mediated BPA-induced downregulation of CatSper subunit expression in GC-2 cells. (**A**) The protein expression levels of CatSper1–4 in Olfr25-overexpressing GC-2 cells after BPA exposure were detected using Western blot analysis. (**B**) Quantitative analysis of the indicated proteins using Image J software. All data are presented as the mean ± SD. ns, not significant. ** *p* < 0.01.

To further clarify the function of Olfr25 in low-dose BPA-induced toxicity, a cell model with stable overexpression of Olfr25 was established. qRT-PCR was used to determine the Olfr25 expression of the cell model. The results showed that *Olfr25* was markedly upregulated after Olfr25 was overexpressed (Figure 5A). qRT-PCR and Western blot analysis were used to detect the expression levels of the pore-forming CatSper subunits after Olfr25 overexpression. The results showed that the expression levels of the CatSper1, CatSper2, and CatSper4 subunits but not the CatSper3 subunit were significantly upregulated compared with the control when Olfr25 was overexpressed (Figure 5B–D). Furthermore, to determine whether Olfr25 mediated the BPA-induced downregulation of CatSper subunit expression, GC-2 cells overexpressing Olfr25 were treated with 80 μM BPA for 48 h. The Western blot results showed that, compared with the vector group, Olfr25 overexpression significantly eliminated the downregulation of the CatSper1, CatSper2, and CatSper4 subunits induced by BPA exposure (Figure 6A,B). Overall, these results indicated that BPA exposure could potentially inhibit the expression of Olfr25, consequently reducing CatSper subunit expression and further lowering intracellular Ca^2+^ levels, thereby eventually inducing sperm dysfunction.

## 4. Discussion

The fertilization of sperm in mammals is an extremely complex and highly coordinated physiological process. Ejaculated spermatozoa need to be stimulated by attractants, such as progesterone and zona pellucida glycoproteins, in the female reproductive tract to find female gametes through chemotaxis and undergo the acrosome reaction before fertilizing the eggs [46,47]. It has been confirmed that Ca^2+^ is a key regulator of many activities, including sperm chemotaxis, capacitation, and the acrosome reaction [13]. Sperm attractants can induce the entry of extracellular Ca^2+^, leading to an increase in [Ca^2+^]i in sperm cells, and then they regulate sperm functions such as the chemotactic response [48]. However, the regulatory mechanisms of sperm chemotaxis and Ca^2+^ levels in sperm are still unclear. The role of ORs in non-chemosensory tissues, especially in the male reproductive system, has attracted increasing attention in recent years [49]. Numerous ORs have been found to be highly expressed in the testis, especially in sperm, which may be crucial in regulating sperm functions, such as spermatogenesis and sperm chemotaxis [26,50]. We found no reports regarding the function and mechanism of *Olfr25*, a newly discovered OR gene. In this study, we found for the first time that interfering with the expression of Olfr25 can significantly reduce [Ca^2+^]i in GC2 cells, suggesting that Olfr25 may participate in sperm function regulation such as chemotaxis by regulating [Ca^2+^]i in mice sperm.

Ca^2+^ is the most important ion among all ions involved in sperm motility. There are several types of calcium channels on the sperm plasma membrane, and they maintain the appropriate concentration of Ca^2+^ in sperm cells [18]. The recognized calcium channels mainly include the Cation Channel of Sperm (CatSper), Transient Receptor Potential Vanilloids (TRPVs), voltage-gated Ca^2+^ channels (VGCCs), and Store-Operated Ca^2+^ Channels (SOCCs) [17,51,52]. In addition to Ca^2+^ channels, the intracellular Ca^2+^ concentration in sperm is also regulated by K^+^-dependent Na^+^/Ca^2+^ exchange (NCKX), plasma membrane Ca^2+^-ATPase (PMCA) and Na^+^/Ca^2+^ exchange (NCX) [53,54,55]. As the most important Ca^2+^ channel, CatSper plays a central role in sperm chemotaxis, capacitation, and the acrosome reaction, and it is essential for successful fertilization in both mice and humans [16,17,47,48]. Several ion channels, such as VGCCs and voltage-gated H^+^ channels (VGHCs), are involved in the activation and modulation of CatSper [18]. Sperm attractants, such as progesterone, zona pellucida glycoproteins, and cyclic nucleotides, can induce the entry of Ca^2+^ into sperm cells through CatSper, thereby promoting chemotaxis and the acrosome reaction [47,56,57]. It has been confirmed that CatSper1–4 are necessary for male fertility and sperm hyperactivated motility [19]. The molecular mechanism regulating the expression of CatSper1–4 in sperm is still unclear. It has been reported that panax ginseng can improve sperm function by inducing CatSper genes and increasing Ca^2+^ levels [58]. CatSper can be directly activated by several odorants to stimulate the entry of Ca^2+^, thereby causing chemotaxis in human sperm [59]. In the present study, we found that knocking down the expression of the OR gene *Olfr25* can significantly reduce the expression levels of CatSper1, 2, and 4, and [Ca^2+^]i. Olfr25 overexpression attenuated the BPA-induced downregulation of CatSper subunit expression. These results suggest that Olfr25 might be involved in sperm function regulation. Further studies are required to uncover the detailed molecular mechanisms by which Olfr25 regulates the expression of CatSper subunits.

As a typical environmental endocrine disruptor, BPA at a high dose is unanimously recognized to induce male reproductive toxicity in rodents and humans [60,61]. However, it remains controversial as to whether BPA at a low dose is toxic to the male reproductive system, and a general consensus cannot be reached. For example, BPA exposure widely ranging from 20 µg/kg to 200 mg/kg was reported to decrease the testicular weight, daily sperm production, and spermatogenesis efficiency in rats [62]. Nevertheless, these alterations could not be observed in a replicated study [63]. Moreover, low-dose BPA exposure (0.2, 2, and 20 µg/kg) was found to cause significant reductions in the epididymal sperm motility and sperm count [64]. Similarly, BPA exposure (2 μg/kg) in adult rats significantly reduced the sperm count and the number of germ cells, which impaired spermatogenesis [65]. In other studies, low-dose BPA exposure (0.2–200 µg/kg or 0.003–5 mg/kg) did not cause changes in reproductive organ weight or in epididymal sperm counts and motility [66,67]. Therefore, studying the reproductive toxicity effects and mechanisms of low-dose BPA exposure is of great significance. The US FDA established a NOAEL of 5 mg/kg/d for humans in 2008 and applied an uncertainty factor of 100 to determine the estimated tolerable daily intake (TDI) of 50 μg/kg/d [68]. However, the European Food Safety Authority (EFSA) lowered TDI 20,000-fold from the prior temporary TDI of 4 μg/kg/d in 2015 to 0.2 ng/kg/d in 2023, emphasizing the risks posed by low-dose BPA [69]. In our study, we converted NOAEL of 5 mg/kg/d for humans to the mouse mouse equivalent dose of 30 mg/kg/d based on the normalization of the dose-to-body surface area [35,36,37]. Concentrations of 0.03, 0.3, and 3 mg/kg/d (representing 1/1000, 1/100, and 1/10 of the dose equal to NOAEL, respectively) were chosen as the low-exposure doses of BPA. Unlike after high-dose BPA exposure, we did not observe the weight of testis, sperm concentration and significant testicular pathological damage after low-dose BPA exposure. However, the chemotaxis, motility, and acrosome reaction of mice sperm significantly decreased after low-dose exposure. In addition, the Ca^2+^ level in mice sperm also significantly decreased after low-dose BPA exposure. These results suggest that low-dose BPA exposure also causes significant male reproductive toxicity mainly by reducing mouse sperm function and fertilization ability.

In this study, we found that the mRNA level of *Olfr25* was significantly downregulated in vivo and in vitro BPA exposure models, indicating a possible mechanism involved in transcriptional regulation. BPA has been reported to bind to the estrogen receptor or androgen receptor and further regulate its downstream response element, finally regulating its targeted genes transcriptionally [70,71]. Further studies are needed to analyze the promoter region of the *Olfr25* gene to elucidate the regulatory mechanism. We further opted GC-2 cells to investigate the mechanism by which Olfr25 regulates the Ca^2+^ signaling pathway. Knockdown of Olfr25 inhibited calcium ion levels and CatSper subunit expression in GC-2 cells. Olfr25 overexpression attenuated the BPA-induced downregulation of CatSper subunit expression in GC-2 cells. Due to the spermatocyte derivation of GC-2 cells, it remains to be further confirmed whether low-dose BPA exposure causes damage to the spermatocyte, preceding spermiogenesis. Our results indicate that Olfr25 is a key target for low-dose BPA-induced sperm dysfunction in mice. Olfr25 may play an important role in low-dose BPA-induced sperm dysfunction by regulating CatSper channel-related molecules and the sperm Ca^2+^ concentration.

## 5. Conclusions

In this study, it was found that low-dose BPA exposure impaired mice sperm function by reducing chemotaxis, motility, and the acrosome reaction. The Olfr25–CatSper-Ca^2+^ pathway may play an important role in low-dose BPA-induced sperm dysfunction in mice. Our results provide data for revealing the function and molecular mechanism of the Olfr25 gene, and they provide a reference basis for the formulation of safe doses of BPA for regulatory agencies.

## Data Availability

The data presented in this study are available upon request from the corresponding author.

## Supplementary Materials

The following supporting information can be downloaded at: https://www.mdpi.com/article/10.3390/toxics12060442/s1, Figure S1: Effect of low-dose BPA exposure on the weight of body and testis in male mice; Figure S2: Effect of low-dose BPA exposure on the sperm concentration in male mice; Figure S3: Effect of low-dose BPA exposure on acrosome reaction of mouse sperm; Figure S4: The effect of Olfr25 knockdown on cell proliferation and cell cycle progression in GC-2 cells; Table S1: The nucleotides used in this study; Table S2: Top 30 GO term annotations; Table S3: Top 30 KEGG annotations.
