# Supplementary material for: The Olfactory Receptor Olfr25 Mediates Sperm Dysfunction Induced by Low-Dose Bisphenol A through the CatSper-Ca2+ Signaling Pathway
Source: Toxics. 2024 Jun 20;12(6):442. doi: 10.3390/toxics12060442 (PMC11209571; doi:10.3390/toxics12060442)
Supplement: Supplementary file 1 [file toxics-12-00442-s001.zip › toxics-2996774-supplementary.pdf]

Supplementary Materials

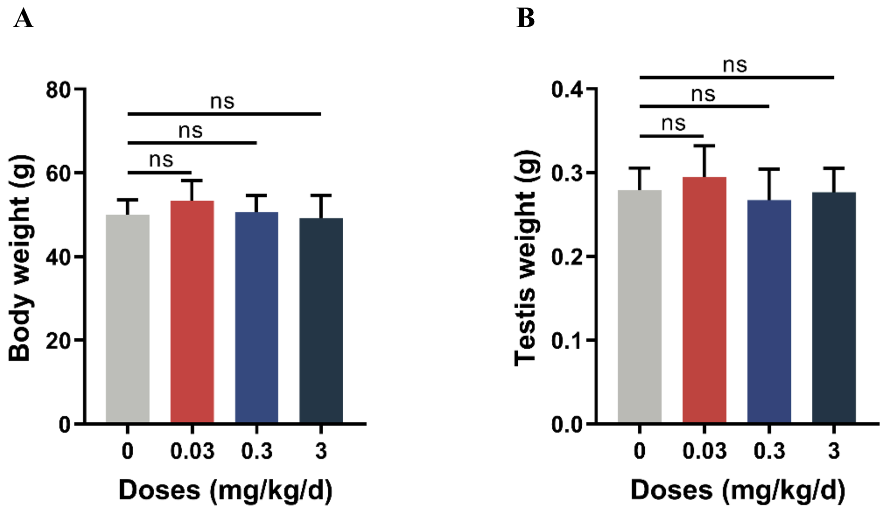

Figure S1 Effect of low-dose BPA exposure on the weight of body and testis in male mice. (A) Body weight of mice (n = 8). (B) Testis weight of mice (n = 8). All data were presented as the means ± SD. ns, not significance.

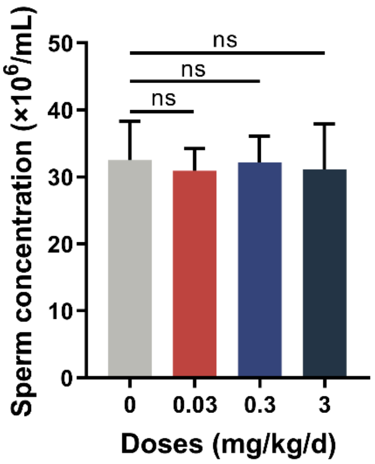

Figure S2 Effect of low-dose BPA exposure on the sperm concentration in male mice. All data were presented as the mean ± SD. n = 8 per group. ns, not significance.

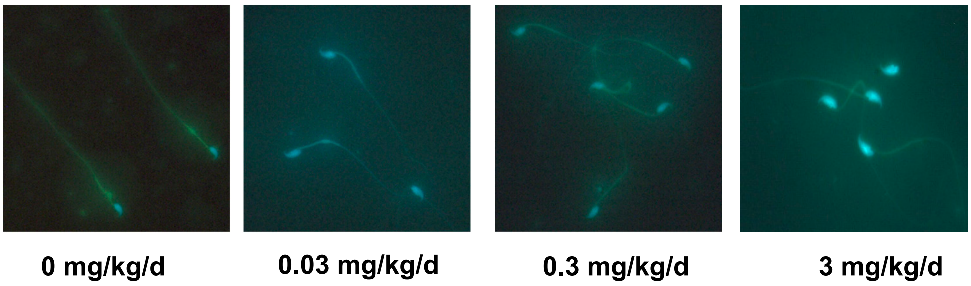

Figure S3 Effect of low-dose BPA exposure on acrosome reaction of mouse sperm. The acrosome reaction of sperm was assessed using chlortetracycline (CTC) and Hoechst 3342 staining. Representative photomicrographs of sperm from male mice exposed with BPA at different doses were shown.

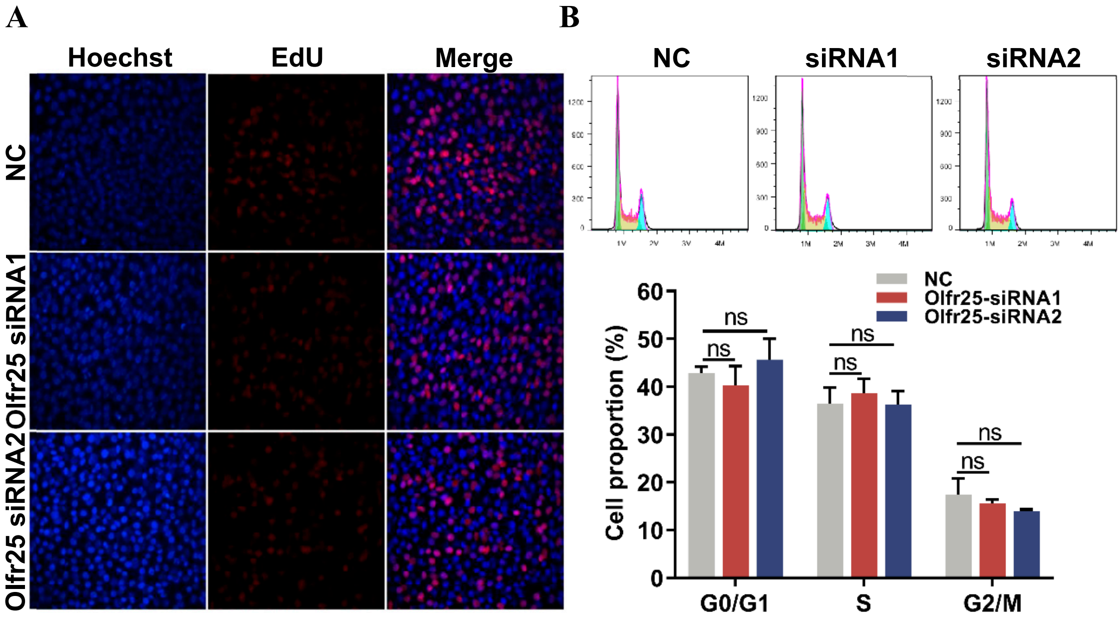

Figure S4 The effect of Olfr25 knockdown on cell proliferation and cell cycle progression in GC-2 cells. (A) The proliferation of GC-2 cells after Olfr25 knockdown was detected by EdU assay. Representative photomicrographs of GC-2 cells labeled with EdU (red) and Hoechst 33342 (blue) were shown. (B) Flow cytometry assays were used to measure the cell cycle distribution of GC-2 cells after Olfr25 knockdown (n=3). The data were presented as the means ± SD. ns, no significance.

Table S1. The nucleotides used in this study.

Name	Sequence (5'→3')	Description
OLFR25-F	TTTGCCCATTCTGAGAGTTGG	Primers for qRT-PCR
OLFR25-R	ACATCAGCAGACAACAGATCCT	
CATSPER1-F	ACCTCTCCATCTCATGTGGG	
CATSPER1-R	CCCTTCACTCAAAGAGGCTG	
CATSPER2-F	AGGTGCCAGAATCTAGCCGTG	
CATSPER2-R	CCTTTCCAAGACTGGTCCCTC	
CATSPER3-F	TGAGCCGGGCGTTTACTATCC	
CATSPER3-R	CAGCCTGTTGACCTCCTCTTGC	
CATSPER4-F	GCATTTGTGCCCAAGCATTTCC	
CATSPER4-R	TTCAGGTGTCCCTCTTCCTCG	
ACTIN-F	GGAGATTACTGCCCTGGCTCCTA	
ACTIN-R	GACTCATCGTACTCCTGCTTGCTG	
NC-siRNA	UUCUCCGAACGUGUCACGU	siRNA for OLFR25
OLFR25-siRNA1	GCAGUAACACCUAUCUCAA	
OLFR25-siRNA2	CCAUGGUUCUGGCAGGUUU	

Table S2. Top 30 of GO term annotation.

Gene Set	Term	Type	Size	P-Value
GO:0008299	isoprenoid biosynthetic process	BP	5	2.146E-06
GO:0016126	sterol biosynthetic process	BP	7	1.889E-07
GO:0006695	cholesterol biosynthetic process	BP	6	1.209E-06
GO:0045071	negative regulation of viral genome replication	BP	4	9.950E-05
GO:0034121	regulation of toll-like receptor signaling pathway	BP	4	2.948E-04
GO:0006720	isoprenoid metabolic process	BP	6	3.701E-05
GO:0005550	pheromone binding	MF	7	1.554E-05
GO:0016125	sterol metabolic process	BP	9	1.965E-06
GO:0000786	nucleosome	CC	6	7.897E-05
GO:0016503	pheromone receptor activity	MF	7	2.757E-05
GO:0008203	cholesterol metabolic process	BP	8	9.638E-06
GO:0048525	negative regulation of viral process	BP	5	3.237E-04
GO:0019236	response to pheromone	BP	7	4.375E-05
GO:0005549	odorant binding	MF	30	2.695E-15
GO:0045069	regulation of viral genome replication	BP	4	1.168E-03
GO:0002224	toll-like receptor signaling pathway	BP	5	4.232E-04
GO:0003725	double-stranded RNA binding	MF	4	1.427E-03
GO:0002221	pattern recognition receptor signaling pathway	BP	6	2.633E-04
GO:0006694	steroid biosynthetic process	BP	8	4.771E-05
GO:0002758	innate immune response-activating signal transduction	BP	6	3.113E-04
GO:0019079	viral genome replication	BP	4	2.074E-03
GO:0002218	activation of innate immune response	BP	6	4.513E-04
GO:0002690	positive regulation of leukocyte chemotaxis	BP	4	2.760E-03
GO:0006334	nucleosome assembly	BP	5	1.333E-03
GO:0050921	positive regulation of chemotaxis	BP	6	5.793E-04
GO:0048520	positive regulation of behavior	BP	7	3.809E-04
GO:0001046	core promoter sequence-specific DNA binding	MF	5	2.252E-03
GO:0045089	positive regulation of innate immune response	BP	7	5.142E-04
GO:0004984	olfactory receptor activity	MF	50	1.918E-17
GO:0006333	chromatin assembly or disassembly	BP	6	1.196E-03

Table S3. Top 30 of KEGG annotation.

Gene Set	Term	Size	P-Value
mmu00100	Steroid biosynthesis	4	3.322e-05
mmu00900	Terpenoid backbone biosynthesis	3	2.735e-04
mmu01040	Biosynthesis of unsaturated fatty acids	3	1.843e-03
mmu00830	Retinol metabolism	4	2.186e-02
mmu05160	Hepatitis C	7	5.423e-03
mmu00500	Starch and sucrose metabolism	2	8.099e-02
mmu04740	Olfactory transduction	44	2.777e-07
mmu00591	Linoleic acid metabolism	2	8.546e-02
mmu05144	Malaria	2	9.006e-02
mmu05218	Melanoma	3	6.802e-02
mmu04620	Toll-like receptor signaling pathway	4	5.810e-02
mmu00980	Metabolism of xenobiotics by cytochrome P450	3	8.339e-02
mmu04623	Cytosolic DNA-sensing pathway	2	1.366e-01
mmu00982	Drug metabolism	3	1.195e-01
mmu04622	RIG-I-like receptor signaling pathway	2	2.188e-01
mmu04662	B cell receptor signaling pathway	2	2.692e-01
mmu04974	Protein digestion and absorption	2	2.843e-01
mmu04380	Osteoclast differentiation	3	2.731e-01
mmu04512	ECM-receptor interaction	2	3.470e-01
mmu05215	Prostate cancer	2	3.797e-01
mmu04060	Cytokine-cytokine receptor interaction	5	4.592e-01
mmu00240	Pyrimidine metabolism	2	4.557e-01
mmu04510	Focal adhesion	4	4.837e-01
mmu05322	Systemic lupus erythematosus	3	4.953e-01
mmu05150	Staphylococcus aureus infection	1	1
mmu05219	Bladder cancer	1	1
mmu05211	Renal cell carcinoma	1	1
mmu03013	RNA transport	1	1
mmu00520	Amino sugar and nucleotide sugar metabolism	1	1
mmu00590	Arachidonic acid metabolism	1	1
